# LONGITUDINAL EVIDENCE FOR A MIDLIFE NADIR IN HUMAN WELL-BEING: RESULTS FROM FOUR DATA SETS*

**DOI:** 10.1111/ecoj.12256

**Published:** 2015-10-15

**Authors:** Terence C. Cheng, Nattavudh Powdthavee, Andrew J. Oswald

**Affiliations:** University of Melbourne; London School of Economics and Political Science and University of Melbourne; University of Warwick

## Abstract

There is a large amount of cross-sectional evidence for a midlife low in the life cycle of human happiness and well-being (a ‘U shape’). Yet no genuinely longitudinal inquiry has uncovered evidence for a U-shaped pattern. Thus, some researchers believe the U is a statistical artefact. We re-examine this fundamental cross-disciplinary question. We suggest a new test. Drawing on four data sets, and only within-person changes in well-being, we document powerful support for a U shape in longitudinal data (without the need for formal regression equations). The article’s methodological contribution is to use the first-derivative properties of a well-being equation.

This article provides what appears to be the first longitudinal (fixed effects) multi-country evidence for a human nadir or midlife ‘crisis’. The background is well known. Human longevity is rising in many nations and there is growing interest in the measurement of well-being in modern society. In what is currently a fast-expanding field at the border between economics and psychology (Easterlin, 2003; Booth and van Ours, 2008; Graham, 2010; Oswald and Wu, 2010; Boyce and Wood, 2011; Carstensen *et al.*, 2011; Benjamin *et al.*, 2012; Diener, 2013), the issue of how people’s happiness and psychological well-being alter over the lifespan is likely to become of increasing scientific interest. This article studies the lives of tens of thousands of randomly sampled individuals over decades and across a number of nations.

A large modern literature by economists and behavioural scientists has documented cross-sectional evidence for an approximately U-shaped path of happiness and well-being over the majority^1^ of the human lifespan (Warr, 1992; Clark and Oswald, 1994; Clark *et al.*, 1996; Frey and Stutzer, 2002; Blanchflower and Oswald, 2008; Booth and van Ours, 2008; Baird *et al.*, 2010; Stone *et al.*, 2010; Lang *et al.*, 2011). This U shape has been found in data from many nations; the work of Di Tella *et al.* (2003), for instance, documents it for each of 12 European countries. An equivalent quadratic life-cycle pattern has recently been reproduced in research on samples of great apes (Weiss *et al.*, 2012). Most recently, Schwandt (2013) has linked the possible idea of a U shape to unmet economic aspirations. This modern research avenue could be viewed as some of the first scientific support for the informal notion – generally attributed to the late Elliott Jaques – of a ‘midlife crisis’ (Jaques, 1965). For a sceptical review of the concept, see Freund and Ritter (2009). The new literature’s findings are in principle relevant to researchers across many fields within the social sciences, medical sciences and the natural sciences.

There are three problems with the published literature:

*Problem 1* is that ‘all attempts to replicate the pattern in genuinely longitudinal data have been a failure’. Prominent among these are two recent studies where no U shape was found (Frijters and Beatton, 2012; Kassenboehmer and Haisken-DeNew, 2012). Ulloa *et al.* (2013) is similarly sceptical. One partial exception is the work of Van Landeghem (2012), who finds evidence consistent with a convex pattern of well-being throughout life but, as he explains, his method is not able to establish a turning point or the existence of a U shape itself.
*Problem 2* is that ‘a number of researchers have argued that the issue of interest is whether in raw unadjusted data, and without regression equations, there is evidence of U shaped well-being through life’. This is the objection, for example of Easterlin (2006) and Glenn (2009),^2^ where they argue that it is inappropriate to use regression equation methods to control for other variables. Their arguments deserve consideration, even though it is a matter of judgment whether it is the raw or adjusted U shape that is of greater scientific interest. Both, in principle, are of importance.
*Problem 3* is that some prominent researchers argue for ‘the reverse of a U shape’, namely, that well-being is actually greatest in midlife. See, for example Easterlin (2006) and Sutin *et al.* (2013). See also the important early paper of Mroczek and Kolarz (1998).

For these reasons, a large multidisciplinary literature currently stands at an impasse. The possibility remains that the U shape is a sheer statistical illusion caused by reliance on cross-sectional data. As De Ree and Alessie (2011) make clear, this is a difficult issue – arguably even an impossible issue – to resolve unambiguously in a regression equation framework, because it is intrinsically hard to estimate models in which the investigator wishes to control for cohort effects, year effects and person fixed-effects. The problem, sometimes called the age-period-cohort or APC problem, is widely recognised. It is the difficulty that by definition *period = year of birth + age*. Thus, a linear regression equation cannot separate the distinct contributions of the three.

## 1. A New Approach

**Fig. 1:**
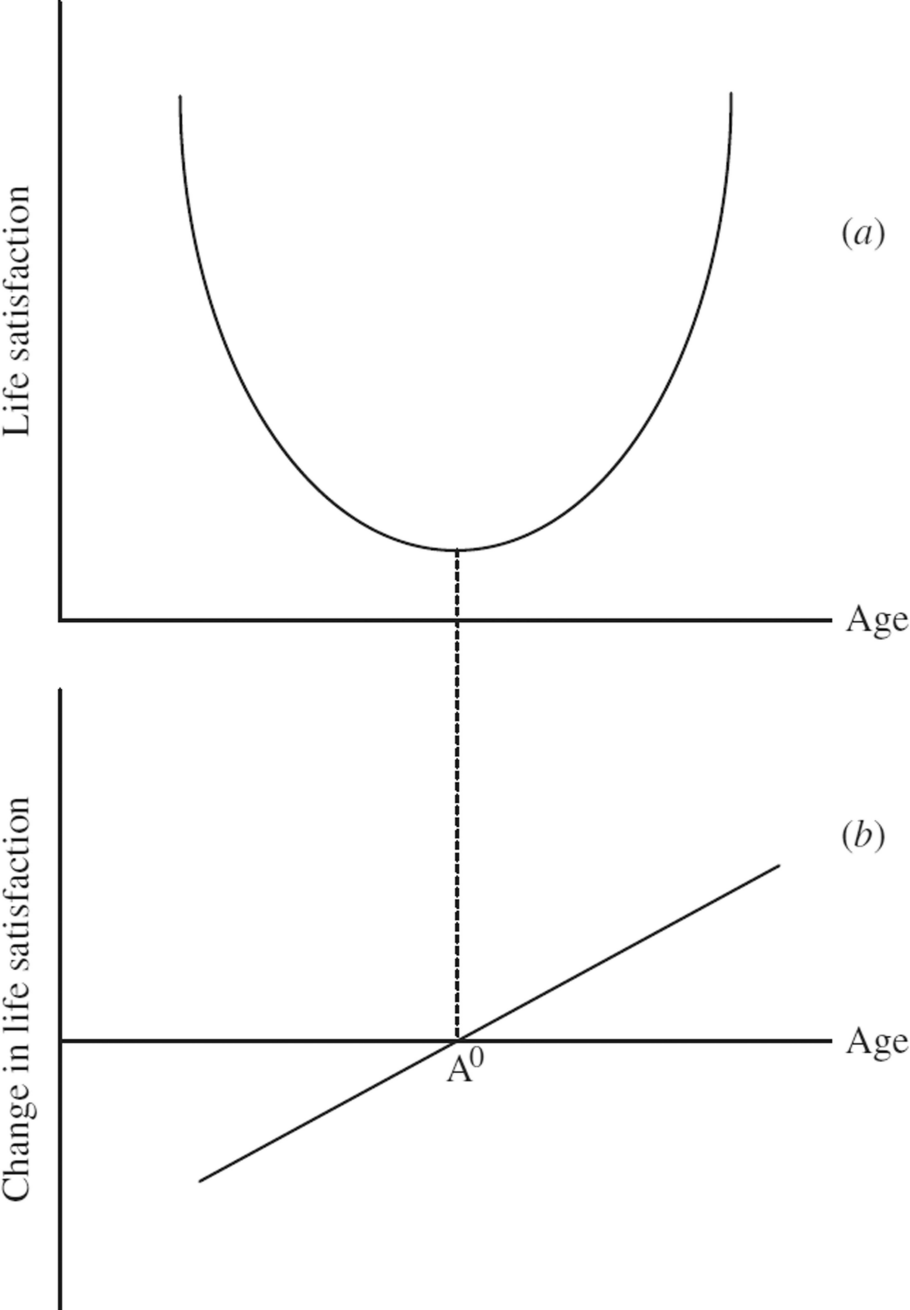
A Non-technical Illustration of the Equivalence Between a U Shape in Life Satisfaction and a Positive Gradient in a Change-in-life-satisfaction Function *Notes*. Starting from low ages, life satisfaction (*a*) decreases, reaches a minimum at age A^0^ and after that increases with age. The change-in-life-satisfaction line (*b*) cuts the horizontal axis (implying zero change) from below at age A^0^ where life satisfaction reaches a minimum. The change-in-life-satisfaction line has a linear and positive gradient with respect to age, corresponding to a U-shaped pattern of life satisfaction over the life cycle. To understand the lower half of this diagram, it is valuable to bear in mind that to the left of A^0^ the change in life satisfaction is negative (i.e. life satisfaction is dropping).

We take a different approach. Our work rests partly upon the ideas of Van Landeghem (2012). We build on the simple mathematical fact that the derivative of a quadratic function is linear. This means that it is possible to test in a different way for the existence of a U shape in human well-being. We illustrate the elementary conceptual idea in Figure 1. The top diagram shows a U shape in life satisfaction, while the bottom diagram shows its derivative, that is, the change in life satisfaction. By elementary calculus, a U shape is equivalent to a linear gradient in the rate-of-change equation. The former is found by integrating back from the latter.

It is, therefore, possible to test for evidence of a U shape in life satisfaction by estimating equations for the change in life satisfaction, as a linear function of a person’s age,^3^ and then examining whether the following hypotheses hold:

- the best-fitting line in a change-in-life-satisfaction equation has a positive gradient with respect to age (which, consistent with a U shape in the level of well-being, would establish convexity of life satisfaction across the age range);
- the best-fitting line in a change-in-life-satisfaction equation begins, at low ages, in the negative quadrant (which would establish that among younger adults the level of life satisfaction is dropping);
- the best-fitting line in a change-in-life-satisfaction equation cuts the horizontal axis in a person’s mid-40s (which would establish that the turning point A^0^ in Figure 1 of the life-satisfaction curve is reached in midlife, and, in conjunction with (*i*) and (*ii*) above, that after that point the level of life satisfaction grows with age).

Together these three results would, if all of them held in the data, establish the empirical existence of U-shaped well-being. They would, as explained, allow the investigator to integrate back, from the rate-of-change equation, to the underlying well-being equation. In testing (*iii*), it is necessary, in principle, to adjust for any underlying annual changes in well-being in the economy and society. Otherwise, the ageing effect might become contaminated by a year effect. It turns out in later analysis, however, that it makes no difference how the adjustment is made.

The kind of test suggested in this article uses information on within-person changes. In our equation, the change in well-being takes the form of a person’s life satisfaction at age A minus that person’s life satisfaction at age A − 1. Such a feature is important. It implies that any results consistent with U-shaped well-being through the life cycle then cannot be attributed to cross-sectional variation between one individual and another. They must stem instead from variation through time in the quality of the lives of the individuals being re-interviewed. Our test could be thought of as a special version of the standard fixed-effects estimator in which, in this case, changes are taken on both sides of the equation but the investigator believes that:

- the right-hand side of the change equation is linear in age; and
- other personal and demographic variables should be omitted.^4^

## 2. Materials and Methods

### 2.1. Data

We used four different data sets covering three countries on individuals up to 70 years of age. Three of the data sets are nationally representative household surveys, namely the British Household Panel Survey (BHPS, 1991–2008), the Household Income and Labour Dynamics in Australia (HILDA, 2001–10) and the German Socio-Economic Panel (SOEP, 1984–2008). The fourth data set comprises a relatively more homogenous sample of medical doctors from the Medicine in Australia Balancing Employment and Life (MABEL) longitudinal study. The average (standard deviation) age of subjects in these data sets is 40.9 years (15.0) for the BHPS, 39.4 years (14.8) for HILDA and 40.5 years (14.5) for SOEP. The average age in the MABEL sample is slightly higher at 45.4 years (11.7).

### 2.2. Measure

Well-being is measured using a conventional life satisfaction questionnaire, which asked all adult individuals in the four data sets: ‘How satisfied are you with your life overall?’ The responses were based on a 7-point scale in the BHPS (1 = ‘very dissatisfied’, …, 7 = ‘very satisfied’), and 11-point scale in the HILDA, SOEP and MABEL (0 = ‘very dissatisfied’, …, 10 = ‘very satisfied’). The life satisfaction question was asked in every survey wave in the HILDA, SOEP and MABEL, whilst it was asked for the first time in Wave 7 of the BHPS, with one year omission in Wave 11. In the eyes of some researchers, particularly those from a psychology background, the single-item nature of our analysis is not necessarily ideal. However, we use large data sets, follow in an earlier tradition of such studies, and use comparisons across a number of data sets as a check on the reliability of results.

## 3. Results

### 3.1. Cross-sectional Evidence

**Fig. 2:**
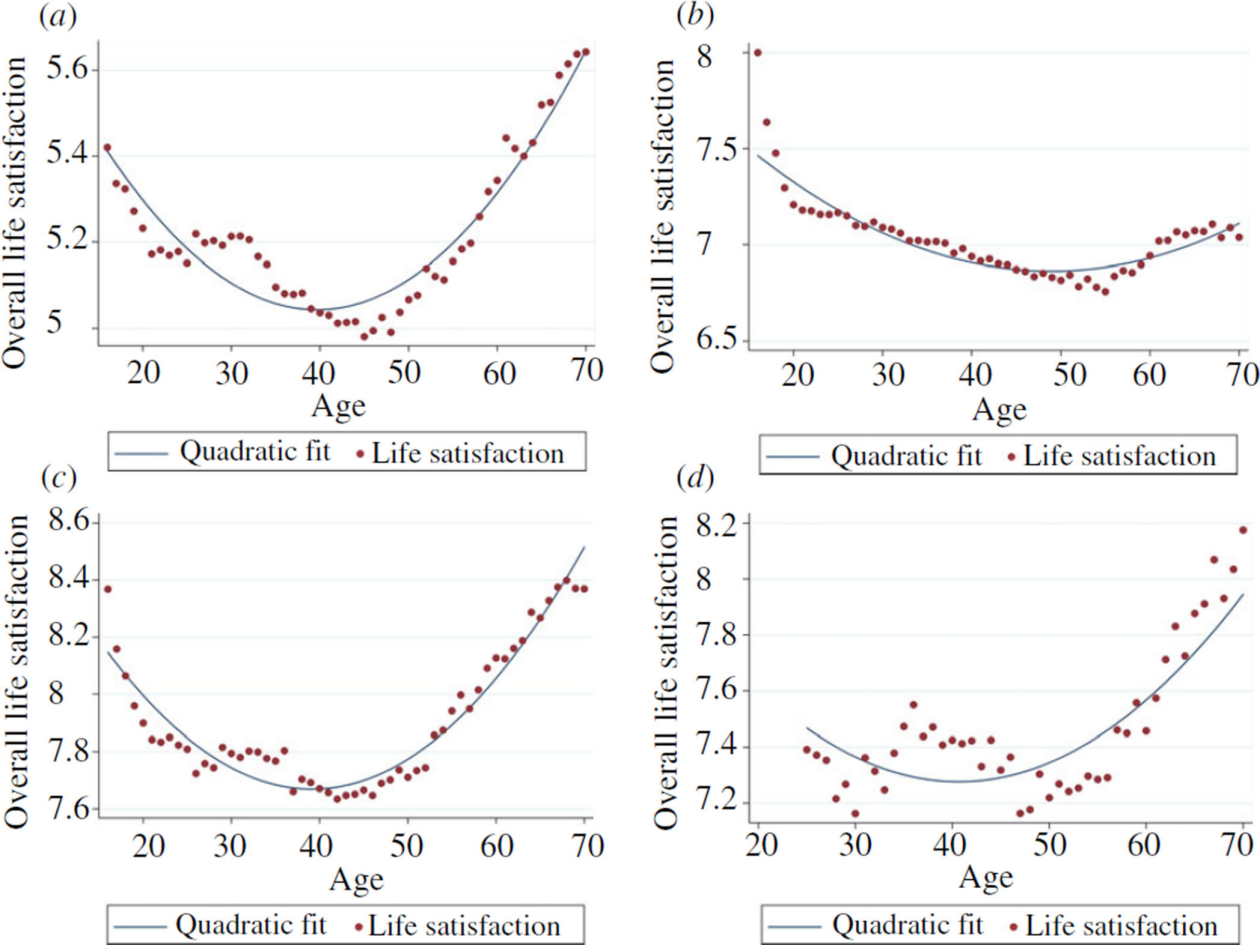
Traditional Cross-sectional Evidence: A U Shape in Life Satisfaction with Age (a) BHPS Data for Great Britain (b) SOEP Data for Germany (c) HILDA Data for Australia (d) MABEL Data for Australia *Notes*. Each dot measures the mean life-satisfaction of individuals of that particular age. The solid curve shows what happens if a quadratic is fitted to the data.

Figure 2 provides cross-sectional evidence for a U-shaped relationship between life satisfaction and age. The Figure is divided into four quadrants – one for each of our four data sets. Figure 2(*a*) is for a random sample of the British population; Figure 2(*b*) is the equivalent for Germany; Figure 2(*c*) is the equivalent for Australia. Figure 2(*d*) is slightly different in its data source. This Figure uses a sample from a single occupation, namely, medical doctors in Australia, which means that the individuals within it are intrinsically more alike than in the other three data sets. Each dot in Figure 2 represents the mean life satisfaction of individuals in the sample of a specific age. The estimate of U-shaped life satisfaction is shown by the fitted quadratic function. The curves’ minima were reached here at respectively ages 43.3 (BHPS), 43.1 (HILDA), 53 (GSEOP) and 40.7 (MABEL), while multiple regression analyses with overall life satisfaction as the outcome variable indicated that linear and quadratic age effects were negative and positive respectively.

We view these findings – depicted in Figure 2 – as reasonably conventional. Hence, we do not dwell on them further but turn instead to a new test that uses within-person changes.

### 3.2. Longitudinal Evidence

**Fig. 3:**
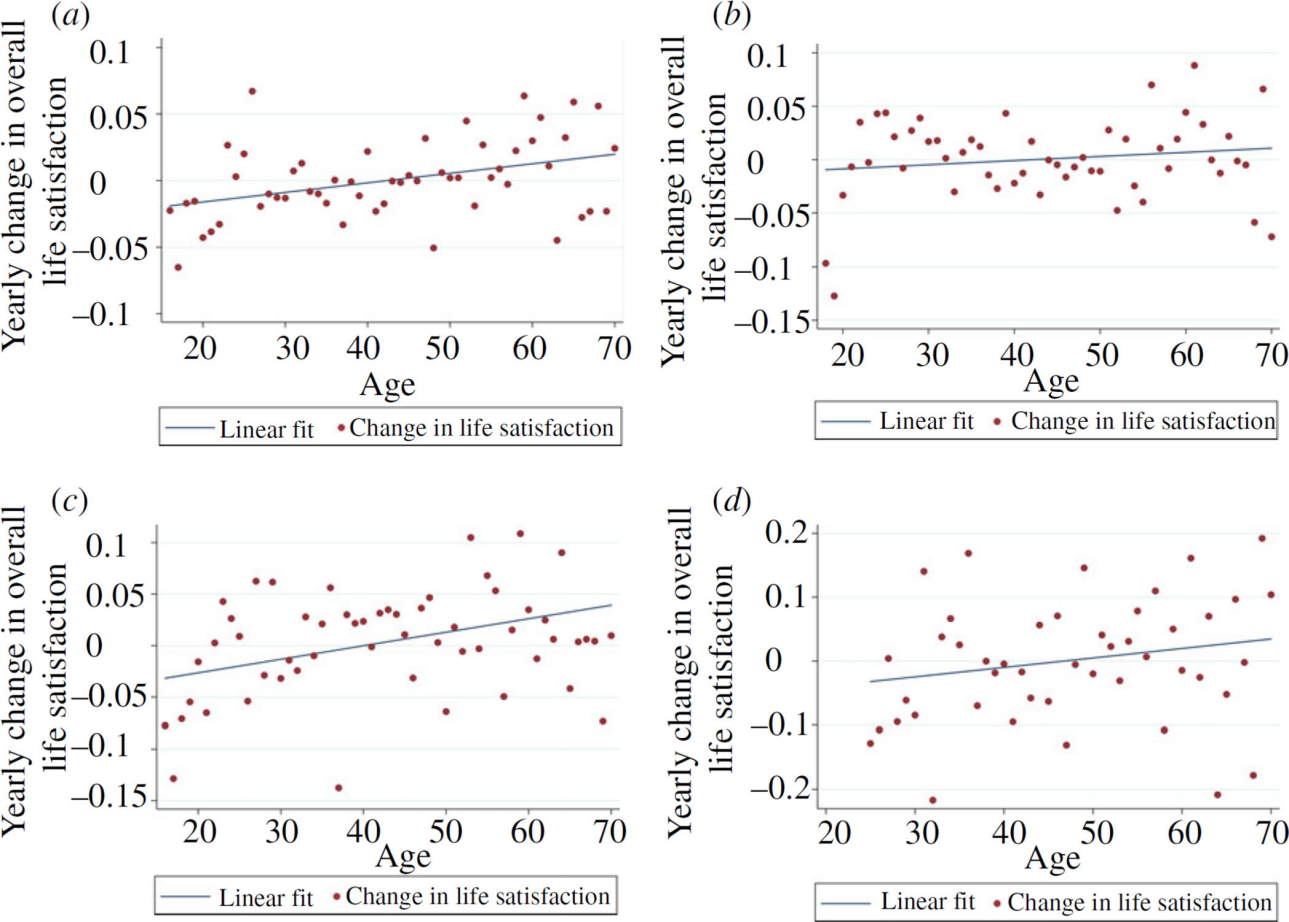
New Longitudinal Evidence: The Gradient of the Change in Life Satisfaction by Age (a) BHPS data for Great Britain (b) SOEP data for Germany (c) HILDA data for Australia (d) MABEL data for Australia *Notes*. Each dot measures the mean change in life satisfaction of individuals of that particular age. The gradients of the first three best-fitting lines are individually significantly different from zero, using a two-tailed t test, at the 99% confidence level. The fourth, for MABEL, is significantly different from zero at the 90% confidence level. In each case, the change in life satisfaction passes through zero (on the *y*-axis) when people are in midlife (on the *x*-axis). This is consistent with, and exactly corresponds to, U-shaped life satisfaction across the ages of individuals in the random population samples. To read these diagrams, it is valuable to bear in mind that to the left of midlife the change in life satisfaction is negative (i.e. life satisfaction is dropping).

Figure 3 presents the study’s key result. Longitudinal evidence for U-shaped well-being across the life cycle is, in effect, demonstrated by the Figure. This emerges in the form of upward-sloping lines, indicating a positive relationship between the change in life satisfaction and age, in each of the four data sets. Figure 3 depicts on the *y*-axis the yearly changes in life satisfaction of individuals from the BHPS, SOEP, HILDA and MABEL. The variable on the horizontal axis is people’s age in years.

Within Figure 3, each dot has a simple interpretation. It is the mean change in life satisfaction of all the people in the sample who are that specific age. For example, in the top-right quadrant, which is denoted Figure 3(*b*) for Germany using the SOEP, the lowest point in the south-west corner of the graph is for Germans, aged 18. It can be seen from the graph that their mean life satisfaction declines that year, namely as they move from 18 to 19, by approximately −0.13 points.

These results provide evidence for the three earlier hypotheses (*i*)–(*iii*) and are thus consistent with U-shaped well-being through people’s lives. The reason is that in each of the four data sets (one for Great Britain, one for Germany and two for Australia), the best-fitting line in Figure 3 begins in the negative quadrant and cuts the horizontal axis from below in people’s 40s. Crucially, that intersection with the *x*-axis is in midlife. The change-in-life-satisfaction function crosses the zero *x*-axis at ages 42.3 in the BHPS, 40.1 in the HILDA, 41.4 in the SOEP and 46.9 in the MABEL. By implication, these are the ages at which well-being reaches a minimum.

It might be thought, although incorrectly, from the figures that the effect of ageing is minor. Because the *y*-axis in these graphs gives the annual rate of change, a number of, say, −0.01 to −0.02 is of consequence. Over a single decade, that would be a decline of 0.1 to 0.2 life satisfaction points, so that over a quarter century – say from approximately age 20 to approximately age 45 – that would be a decline in life satisfaction of a quarter to half of one life-satisfaction point. In size that would become comparable, from a typical regression equation, to a substantial percentage of the effect on well-being of major events such as divorce or unemployment.

Finally, should other independent variables be included? Researchers such as Richard Easterlin and the late Norval Glenn think not. In this article, we have respected that attitude. It is perhaps worth mentioning that if we do include levels of other variables then these longitudinal patterns continue to be robust even after controlling for different socio-economic statuses, including gender, income, employment status, marital status, health and levels of education, as well as the length of time the individual has been in the panel – that is panel conditioning (see the Tables in Appendix A). We are also able to show that the broad results are unaffected by dropping the less satisfied individuals over time. However, we have purposely not estimated, or reported, panel regression equations of the kind employed in some of the recent studies. Our aim in this study has been a different one.

## 4. Further Considerations

It might be felt that this method has an inherent weakness. It might be believed that the constant term in our first-derivative equation is not uniquely identified. Standard calculus, after all, tells us that when we integrate back from a rate-of-change equation there is routinely no way to know the constant term. That would be a serious difficulty for our analysis. The constant is needed to allow us to calculate the cutting points on the *x*-axis in each quadrant of Figure 3.

However, such a concern is incorrect. We circumvent it by using a simple two-step approach. In the first stage, we estimate a regression equation for change-in-life-satisfaction in which the only independent variables are a set of time dummies. Next, we calculate the residual from that equation; this residual is a measure of what might be called the de-trended change in life satisfaction for every person in the data set. We then use the residual as the dependent variable in the change-in-life-satisfaction regression equation that lies behind each quadrant of Figure 3.

Why does this work? Intuitively, it is because the observed changes in people’s life-satisfaction levels have effectively been rescaled relative to the average change in the data in that particular year. The remaining variation in the change in life satisfaction, therefore, stems solely from differences across people, and this allows the correct constant to be calculated. All background economy-wide fluctuations are thus normalised away.^5^

At the request of referees, various other robustness checks were done (the details of these extra findings are available on request from the authors).

First, when we split the data by gender, we obtained similar results for males and females. For example, the cutting points in the equivalents of Figure 3 when done separately for males and females were: in BHPS at age 44 for men and age 43 for women; in HILDA at age 41 for men and age 39 for women; in SOEP at age 44 for men and age 37 for women (though the gradient for women was not quite significantly different from zero); in MABEL at age 50 for men and not well-determined for women. Second, re-estimating Figure 3 with a reduced age range typically replicated the key finding. Because each quadrant of the Figure uses only approximately 55 observations, and the case for dropping observations on the young is itself open to question, we performed only a few experiments with age ranges. Third, we have had put to us, by Richard Easterlin, the interesting hypothesis that a ‘more nuanced’ interpretation of the life-cycle pattern is desirable. It is indeed possible to find some evidence within Figure 2 of a cross-sectional shape that is more complicated than an elementary quadratic. Particularly in some of these quadrants, an argument could be made that there is a kind of early uptick in well-being around the age of, very approximately, 30. That uptick in the data is temporary. The U shape then takes over, once again, as the later 30s approach. But we cannot do this more complex account any real justice, given our data sets, and our simpler, and we think independently interesting, objective. It seems a natural issue for scholars to pursue in future research and would have to be studied in major multi-country data sets. Fourth, another possibility is that there may be a selection effect from the early deaths of unhappy people. This is likely to exist, we agree, and has been discussed before in the literature (Blanchflower and Oswald, 2008) but here we are unable to explore it properly. Because our data range finishes at age 70, we must hope that, in rich long-lived societies of the kind we study in the article, the extent of such selection bias is small. Fifth, a further consideration is to what extent – a reviewer for this JOURNAL has asked us to consider – our method might be helpful in other fields of social or behavioural science. The APC problem has been discussed in many settings (including Glenn, 1976; Fienberg and Mason, 1979; Collins, 1982; Kupper *et al.*, 1985; O’Brien, 2000 and Kuang *et al.*, 2008). However, we feel it is appropriate currently to be cautious about its potential applicability. Because the existing happiness literature has focused particularly on the idea of a quadratic in age, the idea of using our first-derivative method to explore the pure age effect has an obvious attraction. That is because it is trivial mathematically to go from a quadratic form to a linear-gradient test. In more general circumstances, the mathematical transformation is not trivial. Whether this study’s ideas might eventually lead to wider applications in empirical research remains to be understood.

A final point concerns the issue of statistical power. Here, the research literature – across many disciplines – has arguably not been clear enough about the difficulties of the investigative task. If we wish to understand what age does to adult humans, we have, as applied statisticians, less than 100 data points with which to work. It may look at a glance like we have a great deal of statistical power, because we are working with micro data sets, but there is a sense in which we do not, particularly if the scientific aim is to uncover non-linearities. That limits the complexity of any reliable testing procedure. It also makes this a fertile ground, partly because of Type II errors, for different investigators to report (and we suspect, in the future, to keep on reporting) that they have discovered strange things or cannot find well-determined patterns.^6^ Caution – for all of us as researchers – is thus advisable.

## 5. Conclusion

This article provides a new longitudinal test for U-shaped well-being over the majority of the human lifespan.^7^ The intellectual issue is of fundamental importance to social, medical and behavioural sciences.

We use a simple graphical method. We measure the change in well-being of randomly selected individuals each year and then plot that against individuals’ ages. The test is illustrated conceptually in the lower half of Figure 1 and empirically in Figure 3. In this way, we find that, on average, people’s well-being gradually drops until individuals reach midlife. From then on, it picks up smoothly as people go on, in each of three countries and four data sets, to approach the age of 70. Without relying on cross-sectional observations but instead following the same men and women through the years of their evolving lives, we show that there is multi-country evidence for a U shape in the level of human well-being. Claims that the U is an artefact – one perhaps caused by influences such as omitted cohort effects – thus appear to be incorrect.^8^ The article, it should be made clear, deliberately examines unadjusted data and is not subject to the famous APC problem.^9^ We have also here consciously not addressed the issue of what happens to people’s well-being at very high ages (we do not wish to claim that well-being rises into people’s 80s and 90s). That is a different, although interesting, question.

As a reviewer for this JOURNAL has pointed out, it is natural at this juncture to ask for some conceptual model or theory of the U shape. The existence of the pattern requires us to explain both an early fall and a later rise. No standard theory exists in the literature. Nevertheless, it may be helpful to consider the possibility that human wellbeing is characterised by two processes.^10^ Imagine that these occur at the same time within a person (or, to be consistent with Weiss *et al.*, 2012; perhaps even within a non-human primate). Consider them as represented by an additively separable happiness equation: *h*(*a*) = υ(*a*) + *e*(*a*) where *h* is human ‘happiness’, *a* is age, physical vitality is υ(*a*) and emotional maturity is *e*(*a*). Next, assume that each of the two functions is convex from below. The first variable, physical vitality, declines through the life course, and assume it does so at a decelerating rate. The second variable, emotional maturity, rises through life, and assume it does so in an accelerating way. Each of the two constituent curves is monotonic. Then, because the sum of two convex functions is itself convex, the overall well-being function is convex in age. By Rolle’s theorem, the function *h*(*a*) will have an interior turning point in a large class of cases. As an elementary example, let age be defined on the unit interval, vitality be described by the linear function υ = ψ − *a*, and emotional maturity be given by the square of *a*. Then the first derivative of happiness is 2*a* − 1, and happiness reaches a minimum at the mid-point of the age range (namely, at *a* = 0.5). If these two processes, the υ function and the *e* function, are orthogonal to socio-economic forces, then the similarity of regression-corrected and regression-uncorrected well-being curves noted by Stone *et al.* (2010) is actually to be expected.

A U shape in well-being has been reported before in cross-sectional data. By its nature, however, persuasive conclusions about the consequences of human ageing ultimately need longitudinal, rather than cross-sectional, evidence.^11^ We have attempted to provide the first clear evidence.

## Figures and Tables

**Fig. A1:**
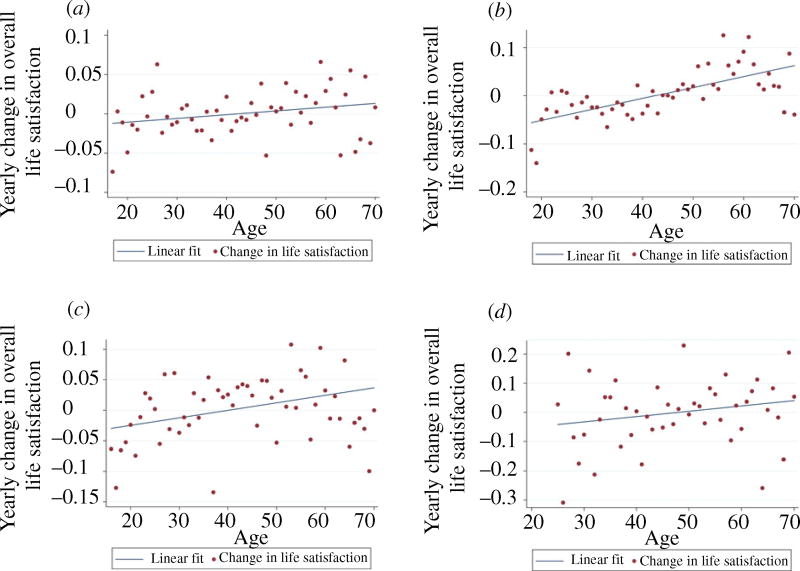
Yearly Change in Life Satisfaction by Age (With Socioeconomic Controls Included in the Regression Equations) (a) BHPS (Intercept = −0.021, p-value = 0.005) (b) SOEP (Intercept = −0.068, p-value = 0.000) (c) HILDA (Intercept = −0.049, p-value = 0.000) (d) MABEL (Intercept = −0.092, p-value = 0.140) *Notes*. In each case, the change in life satisfaction passes through zero (on the *y*-axis) when people are in midlife (on the *x*-axis). This is consistent with, and exactly corresponds to, U-shaped life satisfaction across the ages of individuals in the random population samples.

## Footnotes

1 It is known from prior research that – consistent with common sense – in the last few years of life, as illness occurs, the level of happiness appears to drop off (Blanchflower and Oswald, 2008; Wunder *et al.*, 2013), so over the entire lifespan a more complicated polynomial than a quadratic would be required. Our focus is midlife.

2 Specific emotions, such as anger, are not U shaped or inverted U shaped over the lifespan (Stone *et al.*, 2010). Here, we refer to overall well-being measures.

3 There has also to be a constant. It stems from differentiation of the linear part of the quadratic formula and helps to determine the turning point.

4 They are omitted because the purpose is to look at the unadjusted life-cycle pattern. We are aware that an important scientific question is what age does to well-being after adjusting for other influences.

5 Imagine a country where the average growth of life satisfaction in some particular year is 0.05. Let us say that 35-year olds typically endure a 0.03 drop in life satisfaction as they become age 36, while 55-year olds typically enjoy a 0.03 rise as they become 56. Yet what we would observe in the raw data that year would be a change of +0.02 for 35-year olds and +0.08 for 55-year olds. However, rescaling by exactly 0.05 fixes the problem.

6 It is common in the cross-disciplinary literature on this topic, Ulloa *et al.* (2013) is one recent example, for writers to have a paragraph that says essentially: ‘it is remarkable when we review past writings that in this field some researchers find X, while others find Y, and a few even find Z’.

7 It should be emphasised, not for the whole of the human lifespan. It is not sensible to expect people to be at their happiest close to the point of death. The key issue is instead whether there is a midlife low. A number of researchers who have criticised the U shape have used panel data over the whole lifespan and, in our opinion, this has led, in some cases, to confusion in the literature.

8 We do not doubt, and cannot truly adjudicate on the implications of, the finding of Richard Easterlin that in pooled GSS cross-sections the citizens of the US were, in his data, happiest in midlife. However, column 1 of Table A3 of Oswald and Wu (2011) finds that in modern BRFSS data for the US the age band of lowest life satisfaction is 48–52 years old. This is for a random sample of more than 1 million people using a regression with only age dummies; correcting for other covariates, as in the authors’ column 2 of Table A3, strengthens the evidence for belief in a U shape in today’s US.

9 This claim may sound an unduly bold one. It thus requires clarification. We have not, in truth, ‘solved’ the so-called APC problem. Rather, we have ignored it, or perhaps more accurately we have bypassed it. Our method cannot be used to assess the size of cohort effects and is not designed to do so.

10 There could be more than two; the ideas here will generalise. We would like to record our gratitude to the psychiatrist Dilip Jeste for many helpful discussions on this topic. Professor Jeste argues that what we call the *e*(.) function could, and perhaps should, be thought of as rising wisdom through life.

11 Mention should also be made of two short papers, Clark and Oswald (2006) and Clark (2007), which attempt to argue using BHPS that the U shape is genuine even in longitudinal data. They are downloadable from Andrew Clark’s PSE website.

## Appendix A

### Notes on Accounting for Control Variables, Panel Attrition and First Difference Estimation

Although it is not possible to solve the underlying APC issue, and our contribution should be seen as an analysis of unadjusted data, for completeness we report some further checks.

It is possible to investigate the U-shape pattern in life satisfaction over the life cycle further by regressing within-person change in overall life satisfaction on age of the respondent as follows:

$${\text{LS}}_{\text{it}}-{\text{LS}}_{\text{it}-1}=\Delta {\text{LS}}_{\text{it}}=\beta_{0}+\beta_{1}A_{\text{it}}+\theta {\text{PC}}_{\text{it}}+X_{\text{it}}^{\prime }\gamma +\lambda T_{t}+\varepsilon_{\text{it}},$$

where *i* and *t* index individual and time; *LS_it_* is overall life satisfaction; *A_it_* is individual’s age; *PC_it_* represents panel conditions or the length of time the individual is present in the panel at time *t*; $X_{\text{it}}^{\prime }$ is a vector of standard socio-economic variables such as gender, income, employment status, marital status, health and levels of education (given the homogenous sample in terms of qualifications and employment status of the MABEL sample, the control variables were gender, income, relationship status and health); *T_t_* is time trend; and ε_*it*_ is the error term.

To test successfully whether life satisfaction is U-shaped through life, the following requirements must be satisfied: that

- the slope coefficient on age is positive and statistically significant (β_1_ > 0);
- the function itself is linear; and
- the function cuts the zero *x*-axis in middle age, holding other things constant (β_0_ < 0 and *A_it_* ≅ 40 when Δ*LS_it_* = 0).

Our hypotheses were that, for a robust longitudinal evidence of a perfect U-shaped well-being through life, the year-to-year change-in-life-satisfaction function is linear and will start off negative when the person is young before becoming less negative as he or she goes through life. It then crosses zero *x*-axis when the person is middle aged (around 40 years old) where the function then becomes constantly more positive with age.

The analyses were run using ordinary least squares models that allowed for clustering at the individual level in order to account for the repeated observations of the same individuals in the longitudinal sample.

Graphically, similar ages at the crossing points are obtained if standard socio-economic variables, such as gender, income, employment status, marital status, health and levels of education, are included in the regression equation as additional controls. This is illustrated in Figure A1, where the function now cuts the horizontal axis at the following ages: 42.5 in the BHPS data, 40.1 in the HILDA data, 41.4 in the SOEP data and 48.1 in the MABEL data.

It should perhaps be mentioned that the existence of this midlife nadir is not because of the presence of young children in the household. Adjusting for the number, and the ages, of any dependent offspring leaves the pattern unchanged.

The regressions equations are reported in Table A1. In panel (*a*), the dependent variable is the change in life satisfaction and the independent variable is age and no other controls are included in the equations. Sample sizes vary across the columns from approximately 185,000 observations to approximately 14,000 observations. In the BHPS, HILDA and SOEP data sets, the coefficient on age is positive and is statistically significantly different from zero at the 0.001 level. In the MABEL data, the coefficient is positive but not quite significantly different from zero at the 0.05 level.

**Table A1:** Linear and Quadratic Age Effects on the Within-person Change in Life Satisfaction Between Year t and t − 1

	BHPS		HILDA		SOEP		MABEL	
				
	Coefficient	p value	Coefficient	p value	Coefficient	p value	Coefficient	p value
*Panel (a): time trend, without controls*								
Age	0.001	0.000	0.001	0.000	0.001	0.001	0.001	0.077
*N*	103,159		80,538		185,243		13,910	
*Panel (b): time trend, socio-economic controls*†								
Age	0.001	0.000	0.002	0.000	0.003	0.000	0.002	0.103
Length of time spent in the panel	0.007	0.017	0.036	0.000	0.021	0.000	0.102	0.119
Length of time spent in the panel^2^	−0.0002	0.095	−0.002	0.006	−0.001	0.000	–*	–*
*N*	98,320		80,389		185,169		9,297	
*Panel (c): time trend, socio-economic controls*† *with age-squared*								
Age	0.003	0.059	0.017	0.000	0.006	0.000	0.014	0.138
Age-squared	−0.00002	0.263	−0.0002	0.000	−0.00004	0.010	−0.0001	0.196
Length of time spent in the panel	0.066	0.024	0.028	0.001	0.020	0.000	0.099	0.130
Length of time spent in the panel^2^	−0.0002	0.115	−0.002	0.013	−0.001	0.000	–*	–*
*N*	98,320		80,389		185,169		9,297	

*Notes*. Dependent variable: delta life-satisfaction. Ordinary least squares equations with robust standard errors and clustering at the individual level.

* The squared term for the panel condition variable is omitted because this variable is approximately collinear with the level variable due the short nature of the MABEL panel.

† Socio-economic controls are described in the Section 2.

As an experimental check, we estimated the equation variants described in panel (*b*) and panel (*c*) of Table A1. In panel (*b*), controls are included for how long an individual has been in the panel (to guard against the possibility that someone who has been interviewed over many years acts differently towards a survey interviewer). It can be seen that the results on the age variable are hardly affected. Panel (*c*) switches to allow within the regression specification for a quadratic term in age. This does not alter the substantive findings but, in two cases, HILDA and SOEP, it can be seen that the quadratic term enters statistically significantly in this well-being change equation, which suggests that a perfect quadratic in age in an equation for the level of well-being (as in the modern literature) does not literally match the data. This is an indication that future research may wish to explore more general U-shape polynomials – at least in the case of two of the data sets.

A time trend is included in the regression equations of Table A1. If the alternative case, of a full set of year dummies, is examined instead, the results are essentially identical.

We also explore whether a drop in life satisfaction between *t* and *t* − 1 predicts panel attrition (dropping out from the sample) in *t* + 1 by estimating the following marginal effect probit regression equation:

$${\text{AT}}_{\text{it}}=\pi_{0}+\pi_{1}\Delta {\text{LS}}_{\text{it}}+\pi_{2}A_{\text{it}}+\pi_{3}{\text{PC}}_{\text{it}}+X_{\text{it}}^{\prime }\rho +\sigma T_{t}+\upsilon_{\text{it}},$$

where *AT_it_* denotes dropping out from the sample in *t* + 1. All individuals in the last survey wave – for example, Wave 18 in the BHPS – are excluded from this analysis. Given the short panel of MABEL (3 waves), there is insufficient variation in the attrition model with the panel conditioning variable as one of the explanatory variables. Results for MABEL are thus estimated without the panel conditioning variable on the right-hand side. These results are shown in Table A2. There is some sign that those who are becoming ‘happier’ are slightly less likely to drop out of the sample in the following period, but the broad results are unaffected.

As one further check, we estimated standard kinds of cross-sectional life-satisfaction equations (not reported). These exhibited a well-determined U shape in age across all four data sets. This was done by fitting a quadratic function of life satisfaction through age for all respondents up to 70 years old. The curves’ minima were reached at, respectively, ages 43.3 (BHPS), 43.1 (HILDA), 53 (SOEP) and 40.7 (MABEL), and multiple regression analyses with overall life satisfaction as the outcome variable indicated that linear and quadratic age effects were negative and positive respectively.

**Table A2:** Change-in-life-satisfaction Effect on the Propensity of Dropping out from the Sample in t + 1

	BHPS		HILDA		SOEP		MABEL	
				
	Coefficient	p value	Coefficient	p value	Coefficient	p value	Coefficient	p value
Δ life satisfaction	−0.002	0.004	−0.001	0.077	−0.001	0.020	0.006	0.763
Age	0.0003	0.000	0.0009	0.000	0.001	0.000	−0.006	0.023
Length of time spent in the panel	−0.012	0.000	−0.022	0.000	0.002	0.000	–*	–*
Length of time spent in the panel^2^	0.0003	0.000	0.0009	0.000	−0.0001	0.001	–*	–*
*N*	88,733	70,785		185,169		9,293		

*Notes*. Probit model with robust standard errors and clustering at the individual level. Estimates represent marginal effects. All individuals in the last available survey wave are excluded from the analysis.

* There is insufficient variation in a model of attrition with panel condition variables due to the short nature of the MABEL panel.

**Table A3:** FD Estimation: Life Satisfaction Equations

	BHPS	BHPS	HILDA	HILDA	GSEOP	GSEOP	MABEL	MABEL
								
Variables	LS	LS	LS	LS	LS	LS	LS	LS
*FD estimates: first-differenced regression*								
Age	−0.055*** (0.004)	−0.053*** (0.004)	−0.073*** (0.004)	−0.073*** (0.004)	−0.028*** (0.003)	−0.033*** (0.003)	−0.130*** (0.012)	−0.136*** (0.012)
Age-squared/100	0.067*** (0.005)	0.066*** (0.005)	0.088*** (0.005)	0.090*** (0.005)	0.017*** (0.004)	0.030*** (0.004)	0.139*** (0.012)	0.143*** (0.012)
Time trend		−0.010*** (0.003)		−0.016*** (0.003)		−0.031*** (0.001)		0.082*** (0.011)
Constant	−0.010*** (0.002)	−0.005* (0.003)	0.001 (0.003)	0.002 (0.003)	−0.011*** (0.001)	−0.006*** (0.002)	−0.002 (0.010)	−0.003 (0.010)
Age @ min. point	40.96	41.15	41.14	40.32	82.08	55.09	46.72	47.44
Observations	114,863	114,863	96,795	96,795	349,811	349,811	26,369	26,369

*Notes*.

*** p < 0.01,

** p < 0.05,

* p < 0.1.

Robust standard errors in parenthesis.

Finally, prompted by a reviewer, who asked how our method relates to the statistical literature, Table A3 includes the results when using a traditional FD estimator (Liker *et al.*, 1985; Wooldridge, 2003), which, because it is based on differencing, is analytically the closest to our gradient method. Although not as visually intuitive as our approach, the substantive conclusions from this are similar to those from the graphical analysis of Figure 3. An FD ‘first differences’ estimator has advantages over the ‘within’ fixed-effects estimator in cases where there are autocorrelated errors; it converts a random-walk error term, for example into white noise. Some of the difficulties of estimating non-linear forms using instead the ‘within’ fixed-effects estimator are discussed in the interesting, and perhaps currently relatively under-appreciated, work by McIntosh and Schlenker (2006).
